# Alginate-Encapsulated HDES as a Green Sorbent for Elemental Preconcentration Prior to Determination by FAAS

**DOI:** 10.1021/acsomega.6c01846

**Published:** 2026-07-08

**Authors:** Mateus Olivera Müller, Mariana E. M. Araújo, Adriano Lucas Paiva dos Santos, Floriatan Santos Costa, Marco Tadeu Grassi, Mario Henrique Gonzalez, Clarice D. B. Amaral

**Affiliations:** † Department of Chemistry, 28122 Federal University of Paraná, Curitiba, Paraná 81531-980, Brazil; ‡ Department of Chemistry and Environmental Science, São Paulo State University (UNESP), São José do Rio Preto, São Paulo 15054-000, Brazil

## Abstract

This study presents the development, characterization, and application of a new solid-phase extraction (SPE) sorbent for the preconcentration of Cd, Co, Cu, Fe, Mn, Ni, and Zn in water samples prior to analysis by flame atomic absorption spectrometry (FAAS). The sorbent was prepared by encapsulating hydrophobic deep eutectic solvents (HDES) in calcium alginate, a strategy that prevents HDES accumulation in FAAS and avoids nebulization and transport interferences. The developed method allows the analyte to be eluted into a clean aqueous phase (HNO_3_ 3.5% v/v), containing negligible amounts of HDES. This is demonstrated by the excellent analytical figures of merit obtained, such as the correlation coefficient (*R* ^2^ > 0.9982) and precision (RSD <4.2%), and is further confirmed by the absence of HDES accumulation in the FAAS nebulization system. The encapsulation process involves Methol/Thymol (1:1 molar ratio) emulsified in a sodium alginate solution (10% v v^–1^ HDES content) droplet into a cross-linking agent (CaCl_2_ solution). Under optimized conditions, a 36 mL sample volume (pH 6.0 ± 0.1), 1 g of sorbent, 25 min extraction time, and desorption with 4 mL of 3.5% v v^–1^ HNO_3_ for 5 min were used. In these conditions, the method provided a 9-fold preconcentration factor, with recoveries between 81% and 109% for the seven monitored analytes. The accuracy was confirmed by analysis of real water samples, where concentrations of Cu and Zn ranged from 38 to 74 μg L^–1^ and 68 to 297 μg L^–1^, respectively, showing good agreement (91–109% recovery) with the independent ICP-OES procedure. Additionally, the sorbent demonstrated reusability, highlighting its potential as a low-cost, sustainable, and effective alternative for the trace elements determination in water samples by FAAS.

## Graphical abstract

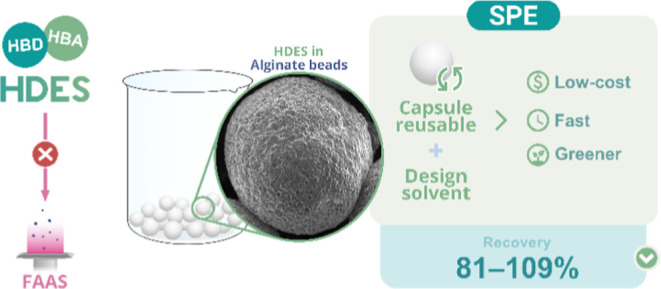

## 1 Introduction

Enhancing sensitivity is a central goal in the development of analytical methods and often drives the search for innovative strategies. Among these, preconcentration techniques stand out as powerful tools to improve detection limits. When paired with the use of environmentally friendly solvents, such as deep eutectic solvents (DES), this combination not only boosts analytical performance but also aligns with the principles of green chemistry. In this context, the integration of DES into sample preparation has emerged as a particularly promising and sustainable approach.^1^

Depending on the components, DESs are considered greener and more sustainable solvents, formed by combining a hydrogen bond acceptor (HBA) and a hydrogen bond donor (HBD).^2^ The strong interaction between HBA and HBD lowers the melting point of the mixture, resulting in a liquid with tunable physicochemical properties.^3^ On the basis of the chosen components, DES can exhibit hydrophobic characteristics and are then referred to as hydrophobic DESs (HDES).^4,5^ HDES have been applied as extractant phases in liquid–liquid extraction (LLE) methods.^6^ However, their use in analytical methods involving sample introduction via nebulization, such as flame atomic absorption spectrometry (FAAS), can be challenging. Matrix, transport effects and changes in the nebulization process may compromise the robustness and reproducibility of the analysis.^7^ These limitations motivated the development of the present approach in which HDES is immobilized within an alginate capsule and employed as a solid-phase sorbent, thereby avoiding the direct introduction of liquid HDES into the FAAS system.

On the other hand, solid-phase extraction (SPE) approaches have demonstrated excellent analytical performance in various sample matrices, especially with the development of new sorbent phases.^8–10^ Sorbents based on alginate have emerged as a promising alternative for designing innovative SPE materials, particularly when combined with HDES.^8^ HDES encapsulated into alginate can significantly enhance extraction efficiency while also contributing to method sustainability.^8^

This combination offers a robust alternative to LLE for trace metal analysis and aligns with current trends in SPE development.^11^ The use of HDES encapsulated in alginate is consistent with the principles of green sample preparation, reducing solvent waste, minimizing toxicological risks, and improving operational safety.^12,13^

In this study, a novel alginate-HDES capsule sorbent was developed for application in SPE and the determination of metals in water samples. The sorbent preparation conditions were optimized, and the experimental parameters for the SPE procedure were refined by using a multivariate experimental design. This approach resulted in significant improvements in method sensitivity and performance using FAAS while also enhancing sustainability as assessed by greenness metrics.

## 2 Experimental Section

### 2.1 Chemicals and Samples

Menthol (Men, 98%) and thymol (Thy, 98%) were supplied by Neon (Brazil), while capric acid (Cap, 98%) and lauric acid (Lau, 98%) were obtained from Synth (Brazil) and used in HDES preparation. Calcium chloride (CaCl_2_) (Alphatec, Brazil), cobalt chloride (CoCl_2_) (Neon, Brazil), and copper sulfate (CuSO_4_) (Synth, Brazil) were used to prepare the cross-linking solutions. Sodium alginate (Sigma-Aldrich, United States) and polysorbate 80 (Tween-80, Neon, Brazil) were used for the emulsion preparation.

Calibration standards were prepared from commercial stock solutions (1000 mg L^–1^, Merck, Germany). Ultrapure nitric acid (HNO_3_) was obtained using a sub-boiling distillation system (model BSB-939-IR, Berghof, Germany). Throughout all the experimental procedures, ultrapure water (18.2 MΩ cm^–1^) was used, obtained from a SynergyUV purification system (Merck, Germany). All materials and glassware were predecontaminated in a 10% v v^–1^ HNO_3_ bath for 24 h.

Water samples were collected from four distinct sources in the city of Curitiba, Brazil. These included river water from the Iguazu River, rainwater and tap water from the Department of Chemistry of the Federal University of Paraná (UFPR), and lake water from the Botanic Garden campus of UFPR. The samples were filtered (0.45 μm) through a pore diameter cellulose acetate membrane (Millipore, Belford, USA), acidified with HNO_3_ to pH < 2.00, and stored under refrigeration at 8 °C. Prior to the application of SPE, the pH of all samples was carefully adjusted to 6.00 ± 0.10. No further processing steps were carried out.

### 2.2 Instrumentation

The SPE procedure was conducted under orbital agitation at 80 rpm by using an orbital shaker (model HMTR, Craltech, Brazil). Sample homogenization was performed with a vortex mixer (Scientific Industries, United States). pH measurements were performed with a pH meter (model HI-2221, Hanna Instruments, Brazil) during sample pH adjustments. Alginate bead sorbent preparation was carried out using a magnetic stirrer (model 752, Fisatom, Brazil) and a peristaltic pump (model IPS-12, Ismatec, Switzerland).

Elemental determination of Cd, Co, Cu, Fe, Mn, Ni, and Zn was performed using a flame atomic absorption spectrometer (model 55B AA, Agilent Technologies, Australia). The flame was generated by using high-purity acetylene gas (99.99%, White Martins, Brazil) and compressed air. The slit width was set to 1 nm, and background correction with a deuterium lamp was applied for all elements, using an oxidizing flame with acetylene and air flow rates of 1.50 L min^–1^ and 6.50 L min^–1^, respectively.

### 2.3 HDES and Alginate Sorbent Preparation

The HDES was prepared using a microwave-assisted heating system, as previously described.^14^ The HBA and HBD compounds were accurately weighed at the specified molar ratio and mixed under vortex agitation in a closed vessel. The mixture was then subjected to microwave-assisted heating for 30 s in two consecutive cycles.

The sodium alginate suspension (1.0% w v^–1^) was prepared in water and stirred until fully solubilized. Subsequently, polysorbate 80 (0.10% v v^–1^) and HDES (Men/Thy, 1:1 molar ratio) were added to the alginate suspension at a concentration of 10% v v^–1^ to form a stable emulsion. The alginate-HDES emulsion was then dispensed using a peristaltic pump (80 rpm) and dripped at a rate of approximately 3 mL min^–1^ into a cooled (4 °C) CaCl_2_ solution (0.20 mol L^–1^), which acted as the cross-linking agent. The system was stirred at 200 rpm. The resulting beads were kept in the cross-linking solution for 60 min, filtered, and then rinsed in diluted HNO_3_ (1% v v^–1^) for 10 min. Finally, the beads were filtered again, rinsed with ultrapure water, and placed on a Petri dish to dry at room temperature until constant mass.

### 2.4 Sorbent Characterization

The morphological properties of the synthesized sorbent were investigated by using scanning electron microscopy (SEM). Morphological analysis was performed by using a Tescan Vega3 LMU microscope (Tescan Orsay Holding, Czech Republic) operating at an acceleration voltage of 15 kV. Prior to analysis, samples were sputter-coated with a thin gold layer to improve the conductivity and resolution. The FTIR characterization was conducted using an Alpha II FT-IR spectrometer (Bruker, Germany) operating with a spectral range of 350 to 4000 cm^–1^ and resolution of 0.75 cm^–1^.

### 2.5 Multivariate Experimental Optimization

The experimental parameters involved in the extraction and desorption steps were optimized using an experimental design. For the extraction step, a Box-Behnken design (BBD) was employed to optimize the sorbent mass (200, 600, and 1000 mg), sample pH (2.0, 4.0, and 6.0), and extraction time (5, 15, and 25 min). Also, a Doehlert design (DD) was applied to optimize the desorption time (1, 3, 5, 7, and 9 min) and the HNO_3_ concentration used for the desorption step (1, 3, and 5% v v^–1^).

The experiments were performed in random order, and a multiple response (MR), calculated from the % recoveries of the analytes (Cd, Co, Cu, Fe, Mn, Ni, and Zn) obtained in each experiment, was employed as the experimental response.^15^ The fitted mathematical models were evaluated using an analysis of variance (ANOVA). The robustness of the statistical models was confirmed on the basis of the significance of the regression, the lack-of-fit test, and the coefficient of determination (*R* ^2^). All data analyses were performed using Statistica software (version 12, StatSoft, United States) at a 95% confidence level.

### 2.6 SPE Procedure

The alginate-HDES bead sorbent was used in the SPE method applied to water samples. Alginate-HDES beads (1.0 g) were added to the sample (36 mL), and analyte extraction was carried out for 25 min under orbital shaking. Subsequently, the residual sample was discarded, and 4.0 mL of HNO_3_ (3.5% v v^–1^) was added, allowing a contact time of 5 min for analyte desorption. The sorbent phase was then removed, and the enriched solution was analyzed using FAAS. Additionally, it is important to note that the volumes of enriched solutions in each analysis are constrained by instrumental limitations. In the case of FAAS, due to its monoelemental nature, a minimum volume of 0.5 mL is required per measurement, which necessitates a total of 4 mL to analyze the seven monitored elements in a single run. However, depending on the analytical goals, smaller desorption volumes may be employed to achieve higher enrichment factors (EFs) at the expense of a reduced number of monitored analytes per determination.

### 2.7 Analytical Performance

To evaluate the accuracy of the SPE procedure, two validation approaches were employed: comparison with an independent procedure and the standard addition method. First, the results obtained using the proposed procedure were compared with those from conventional acid digestion (CAD) followed by ICP-OES determination. All four samples (tap, rain, lake, and river water) were processed using both procedures. In addition, a standard addition test was performed by spiking one sample from each water sample at three concentration levels, 100, 200, and 300 μg L^–1^. The samples were then prepared using the SPE procedure, and accuracy was determined based on percent recovery within the acceptable range from 80 to 110%.

Precision was checked using both intraday and interday relative standard deviation (RSD). Intraday precision was assessed by analyzing four replicates of spiked samples at 500 μg L^–1^ within a single analytical run. Interday precision was determined by repeating the same procedure over three consecutive days. The limit of detection (LOD) and limit of quantification (LOQ) were calculated as 3σ/A and 10σ/A, respectively, where σ is the standard deviation of ten blank measurements and *A* is the slope of the calibration curve. Also, the procedure applied for LOD and LOQ calculation was performed for each analyzed element individually with the desorption step using 0.5 mL of nitric acid solution and the respective monoelemental analysis.

## 3 Results and Discussion

### 3.1 Sorbent Composition

Before optimization of the SPE application, it is essential to refine the composition of the solid phase itself. This procedure ensures not only improved analyte extraction efficiency but also the production of stable and reproducible sorbent alginate-HDES beads. The optimization process was carried out, using a univariate approach, in three stages: selection of the cross-linking ion, evaluation of the HDES in alginate beads, and the alginate/HDES ratio in the emulsion.

The first optimization focused on the cross-linking ions calcium (Ca^2+^), cobalt (Co^2+^), and copper (Cu^2+^) used for the alginate gelation process. Although all three ions yielded mechanically stable sorbents, only Ca^2+^ was selected as the optimal cross-linker for two reasons. First, unlike Co^2+^ and Cu^2+^, calcium is not an analyte in this work and therefore does not interfere in the determination. Additionally, the alginate beads cross-linked with Ca^2+^ demonstrated the best MR and the smallest deviations between replicates. The data are presented in Figure S1 (Supporting Information).

The next optimization step involved testing different HDES compositions and HDES compound combinations, including capric acid/lauric acid (Cap/Lau), capric acid/thymol (Cap/Thy), menthol/capric acid (Men/Cap), menthol/lauric acid (Men/Lau), menthol/thymol (Men/Thy), and thymol/lauric acid (Thy/Lau). Among these, Thy/Lau-based beads proved to be unstable and deteriorated during the drying process. The remaining bead formulations were assessed based on the recovery efficiency after SPE application, with Men/Thy exhibiting the highest % recovery, as shown in Figure 1A, and thus being selected for further studies.

**Figure 1:**
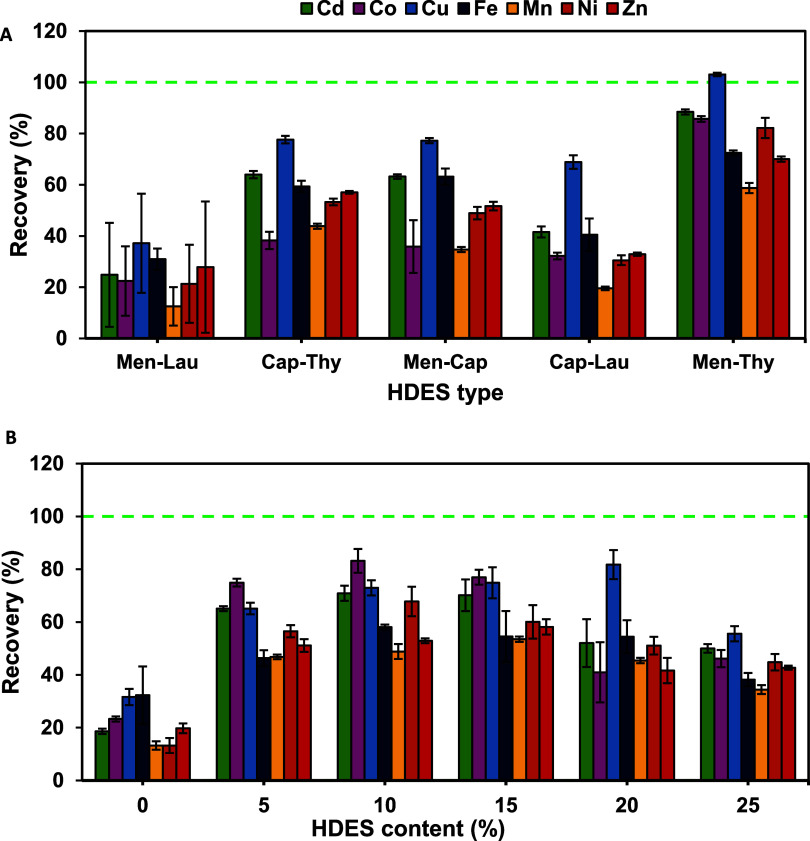
Recovery (%) obtained using different HBA/HBD combinations in the encapsulated HDES (A). Effect of the amount of HDES in the emulsion on the %recovery of analytes (B).

Finally, the volumetric proportion of the sodium alginate solution to HDES in the emulsion was optimized. Different volume ratios of 75, 80, 85, 90, 95, and 100% of sodium alginate solution and the remainder being HDES were evaluated. The 90% alginate formulation achieved the highest MR, as shown in Figure 1B. The beads composed solely of alginate (100%) demonstrated poor analyte recovery, emphasizing the critical role of the HDES component in the effectiveness of extraction.

Following composition optimization, Ca^2+^-cross-linked alginate capsules containing 10% HDES (Men/Thy) were established as the optimal sorbent formulation, demonstrating mechanical stability and superior extraction efficiency for the target analytes.

### 3.2 Sorbent Characterization

The morphological properties of the synthesized alginate-HDES beads were investigated using SEM to verify the efficiency of the encapsulation process and the structural features relevant to the extraction performance, and FTIR analysis was used to demonstrate the successful encapsulation of the HDES and its chemical stability. As shown in Figure 2A,B, the sorbent exhibited a spherical morphology with a rough surface. This textural characteristic is analytically advantageous, as the increased surface roughness enhances the contact area between the sorbent and the aqueous sample.

**Figure 2:**
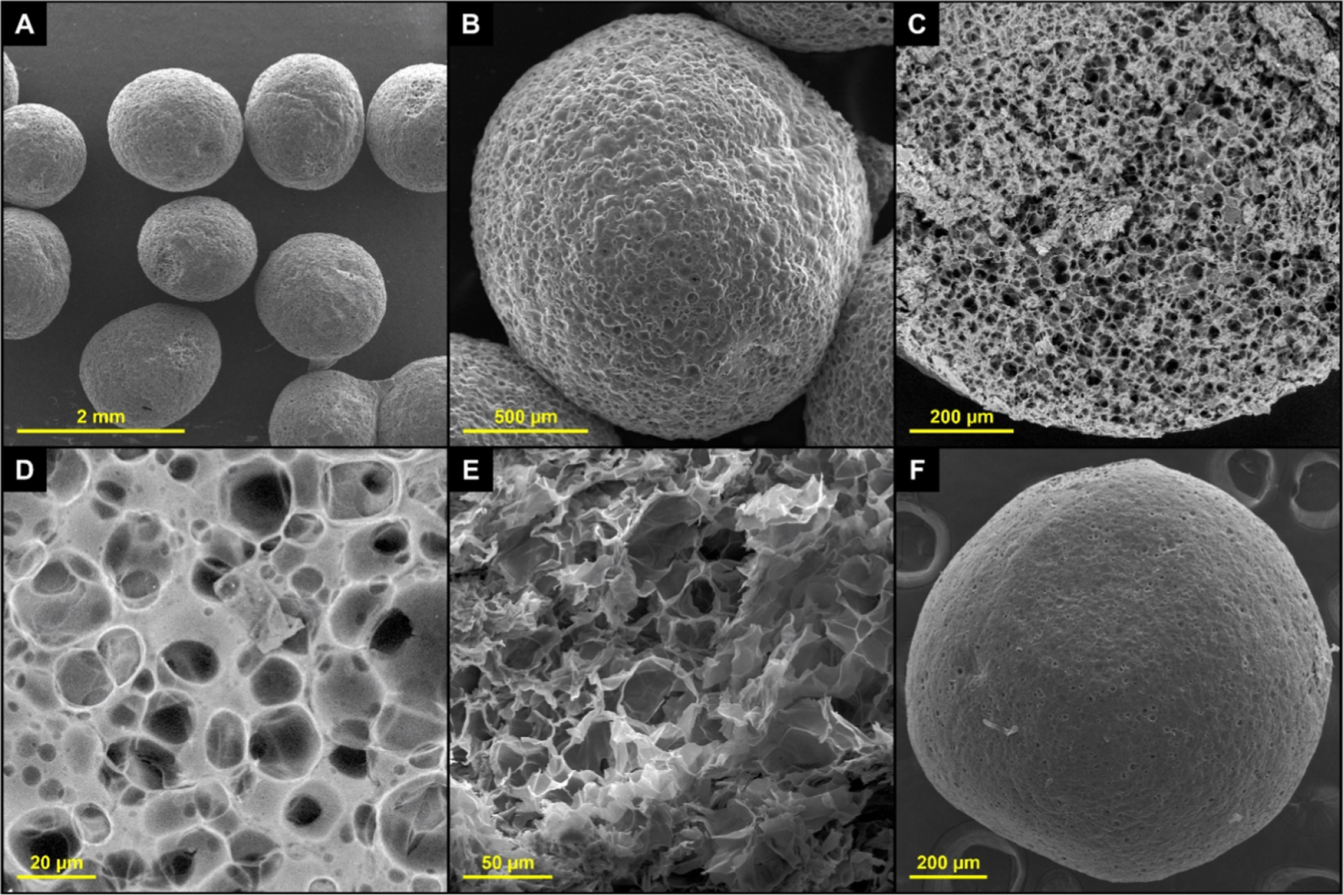
SEM images of alginate beads (A) and details of the bead surface structure (B). Cross-section of the sorbent containing HDES (C) and visualization of HDES pockets within the alginate bead structure (D) and cross-section of the sorbent with the HDES removed (E). Visualization of the alginate bead after four application cycles involving cleaning, extraction, and desorption steps (F).

The cross-sectional analysis in Figure 2C reveals a heterogeneous internal structure where the HDES is dispersed within the alginate matrix. A critical function of the calcium alginate network is to prevent the HDES from aggregating, keeping the HDES dispersed as small pockets, as shown in Figure 2D, and the alginate ensures that a high active surface area is maintained while allowing the diffusion of aqueous samples for analyte interaction. Furthermore, the visualization of the structure after the HDES removal, as shown in Figure 2E, corroborates the existence of the alginate network that retains the extracting phase.

Mechanical integrity is necessary for minimizing the HDES loss and ensuring the reproducibility of the SPE procedure. In related works employing the extrusion dripping method to produce alginate materials with hydrophobic components, similar structures are observed.^16,17^

The FTIR analysis Figure S2 (Supporting Information) serves as proof of HDES encapsulation. The spectrum exhibits two distinct peaks at approximately 2925 cm^–1^ and 2855 cm^–1^, attributed to the asymmetric and symmetric C–H stretching vibrations of the alkyl groups (−CH_2_ and −CH_3_) from the menthol and thymol components.^18^ These characteristic bands are absent (or negligible) in pure calcium alginate.^19^ The presence of these C–H stretching bands in the sorbent spectrum serves as evidence that the HDES was successfully trapped into the sorbent structure.

### 3.3 SPE Experimental Conditions

The SPE optimization process was performed to evaluate the extraction and desorption steps. For the adsorption, a BBD was employed to evaluate extraction time (A), sample pH (B), and sorbent mass (C). The data set used in the model fitting is shown in the Supporting Information (Table S1).

The low pH range was selected based on the sorbent instability in alkaline media. The statistical analysis, ANOVA (Table S2), revealed that quadratic models demonstrated significant regression (*F*-calculated = 70.8, Df = 9.5 > *F* critical value = 4.77) with satisfactory fit (*p* < 0.001) and high descriptive capacity (*R* ^2^ = 0.992). The contour plot in Figure 3A–C allows us to identify conditions that maximize analyte extraction (1000 mg of sorbent, sample pH of 6, and 25 min of extraction).

**Figure 3:**
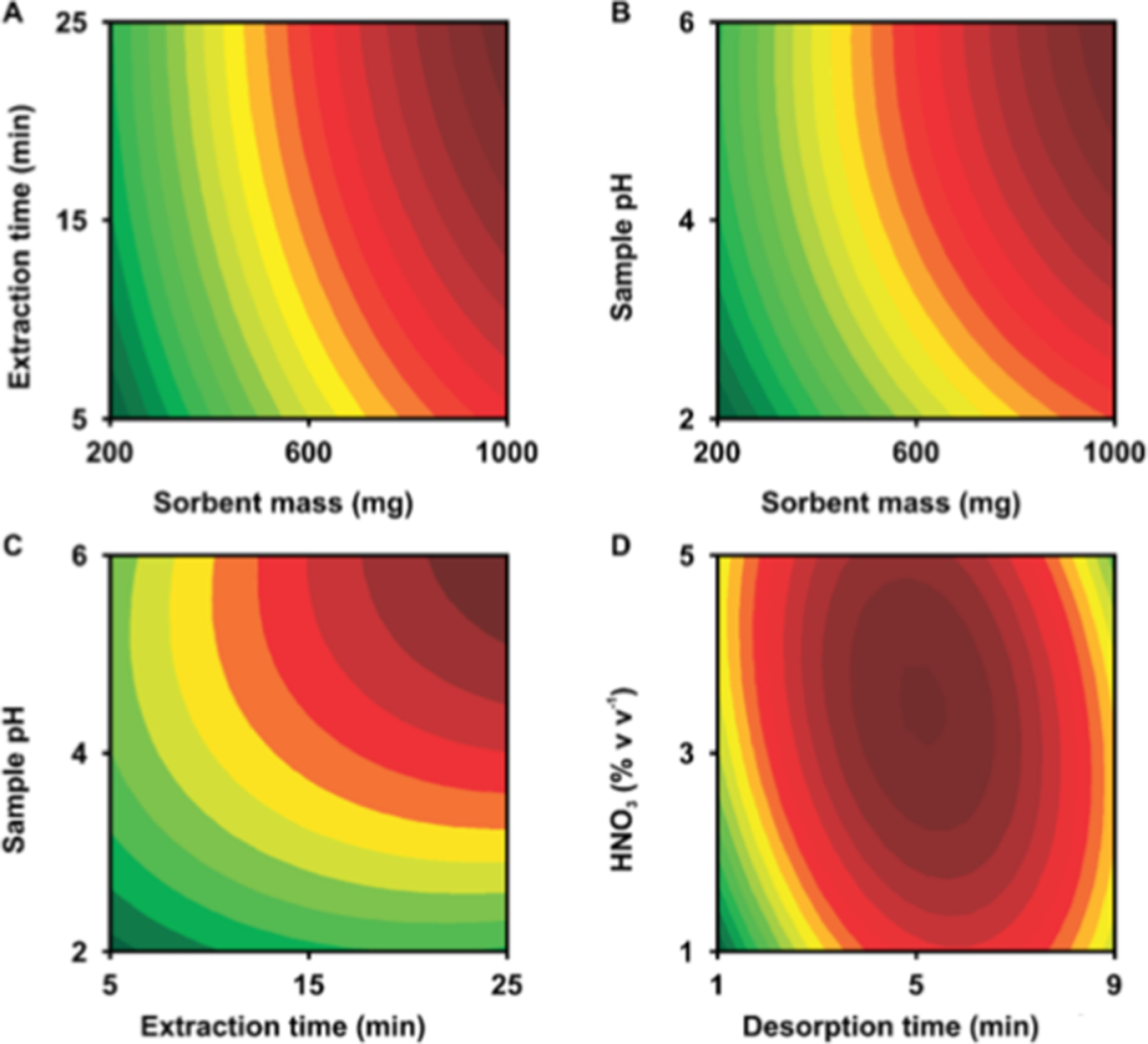
Contour plots generated from the quadratic model obtained via the Box-Behnken design (A–C) and contour plot generated from the quadratic model obtained via the Doehlert design (D), both using multiple responses as the experimental response.

Chemically, the optimization results indicated that all variables tended toward their maximum values within the experimental domain. The need for a longer extraction time (25 min) suggests that the process is influenced by mass transfer effects, likely due to the time required for analytes to diffuse through the alginate matrix and reach the HDES pockets. Similarly, the requirement for a higher sorbent mass (1000 mg) is justified by the increase in the number of available interaction sites, ensuring enough capacity to retain all of the analytes. Regarding the sample pH, the optimal value of 6.0 indicates that more acidic conditions (lower pH) increase the solubility of the analytes and promote competition with the sorbent, thereby lowering the efficiency of the extraction. Consequently, the combination of maximum time, mass, and pH 6 results in an experimental condition that provides a high extraction capacity.

Following the adsorption step, the desorption conditions were optimized using a DD with the optimal conditions from the previous study. The variables monitored were desorption time (1 to 9 min) and HNO_3_ concentration (1 to 5% v v^–1^), aiming for the minimal elution time and acid concentration required for analyte recovery at acceptable levels. The data set used in the modeling is presented in Supporting Information (Table S3). This approach ensured efficient desorption while minimizing potential damage to the sorbent. The statistical analysis, ANOVA, revealed that quadratic models demonstrated significant regression (*F*-calculated = 58.1, Df = 5.3 > *F* critical value = 9.013) with satisfactory fit (*p* = 0.004) and high descriptive capacity (*R* ^2^ = 0.990).

The contour plot in Figure 3D identifies the best desorption conditions where analyte extraction is higher (desorption for 5 min and 3.5% v v^–1^ HNO_3_). The interpretation of the desorption data reveals a critical balance between efficiency and sorbent stability. While the acid concentration is relevant for the protonation and removal of analytes, higher HNO_3_ concentrations were observed to lead to lower recoveries. This behavior is likely due to the accelerated degradation of the alginate bead structure under highly acidic conditions, which can cause an increased leaching of the HDES phase. Similarly, prolonged desorption times also exhibited a similar effect, as extended exposure to the acidic medium promotes further structural degradation, which can lead to an increase in matrix effects, reducing the analytical signal. In contrast, low acid concentrations and short contact times were insufficient to promote the complete desorption of the analytes. Therefore, the optimized conditions (3.5% HNO_3_, 5 min) represent a compromise where the desorption efficiency is maximized while preserving the structural integrity of the sorbent.

The last step was to combine the ideal conditions from both experimental designs and then apply larger volumes of samples (24, 36, and 48 mL) to obtain higher preconcentration factors (6, 9, and 12, respectively). The results of this study are shown in Figure S3 (Supporting Information). A decrease in the recovery was observed at the highest volume tested (48 mL). This behavior is attributed to the limitation imposed by the fixed sorbent mass (1.0 g); increasing the sample volume beyond 36 mL creates an unfavorable volume-to-mass ratio, leading to the saturation of available binding sites and preventing the complete retention of the total analyte load. Consequently, 36 mL was selected as the optimal volume.

This comprehensive optimization strategy addressing both sorbent composition and extraction parameters resulted in a robust, reproducible SPE method with recoveries ranging from 87 to 108%, with a 9-fold preconcentration factor, with the following conditions: 36 mL of the sample, pH 6, 25 min of extraction with 1.0 g of sorbent, and desorption for 5 min with 4 mL of HNO_3_ (3.5% v v^–1^).

### 3.4 Reutilization Study

The reusability of the HDES-alginate sorbent was evaluated through consecutive SPE cycles under optimized conditions. Between each extraction, the sorbent underwent the regeneration process, where it was washed with HNO_3_ solution (1.0% v v^–1^), followed by rinsing with ultrapure water and then dried for subsequent use. The recoveries obtained are presented in Figure 4. The satisfactory recovery obtained for all analytes during the second use demonstrates that the degradation resulting from the first use of the material is negligible, as both uses achieved recoveries within the acceptable range of 80–110%. However, upon the third application, a decline in recovery rates was observed, accompanied by an increase in the standard deviation of the results. This resulted in unsatisfactory recoveries (<80%) for Fe, Mn, Ni, and Zn, followed by Co in the subsequent use. It is important to highlight that the HDES loss resulting from material degradation, even after four uses, is tolerated by the FAAS technique and does not lead to undesired HDES buildup in the system.

**Figure 4:**
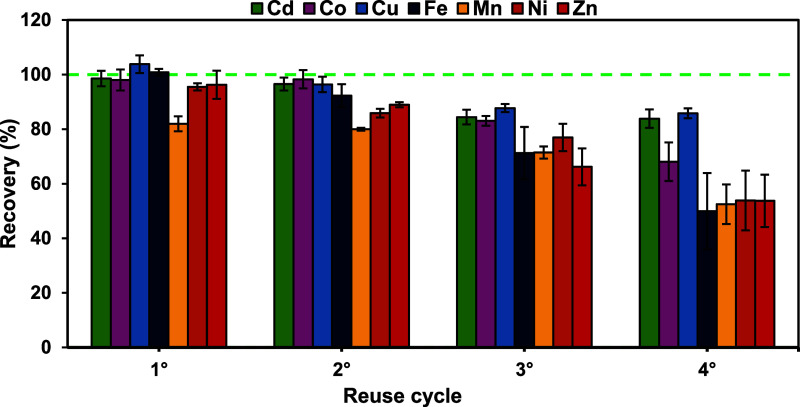
Recovery (%) of the proposed method with sorbent reuse over four cycles, following the regeneration step between each use.

From the SEM images shown in Figure 2B and F, it is possible to observe the degradation of the solid phase after four cycles of use. The visible alteration of the HDES/alginate structure corroborates the results obtained in Figure 4, confirming that the structural instability is a limiting factor for long-term reusability.

However, this limited reusability does not hinder the applicability of the method. The proposed sorbent was designed as a cost-effective disposable material. A cost analysis estimated the synthesis cost to be less than USD 0.15 per gram. Consequently, the tradeoff between the limited cycling capacity and the low economic and environmental cost of synthesis supports the sorbent’s viability as a disposable or short-term-use material, aligning with a sustainable single-use approach for routine analysis.

### 3.5 Analytical Performance

The accuracy of the developed procedure was evaluated through two distinct approaches. Standard addition tests were performed at three concentration levels for all water samples. As presented in Table 1, the recoveries obtained were consistently within the acceptable range of 80–110%, confirming the absence of significant matrix effects and validating the suitability of the method for different water types.

**Table 1:** Evaluation of Method Accuracy through a Standard Addition Test at Three Analyte Concentration Levels^a^

	level	Cd		Co		Cu		Fe		Mn		Ni		Zn^b^	
Sample	(μg L^–1^)	(μg L^–1^)	rec (%)	(μg L^–1^)	rec (%)	(μg L^–1^)	rec (%)	(μg L^–1^)	rec (%)	(μg L^–1^)	rec (%)	(μg L^–1^)	rec (%)	(μg L^–1^)	rec (%)
lake water	0	<LOD	-	<LOD	-	38.3	-	<LOD	-	<LOD	-	<LOD	-	296.9	-
	100	107.5	108	96.0	96	135.4	97	82.0	82	100.2	100	98.4	98	367.0	105
	200	207.5	104	184.1	92	237.3	99	164.1	83	180.1	90	174.6	87	413.6	88
	300	321.4	106	288.0	96	318.2	93	287.9	94	246.0	82	263.8	88	460.1	82
rainwater	0	<LOD	-	<LOD	-	42.6	-	<LOD	-	<LOD	-	<LOD	-	80.9	
	100	100.0	100	102.9	103	139.1	97	83.3	83	102.6	103	107.4	107	148.0	100
	200	216.2	107	183.1	90	238.0	98	162.3	81	170.9	85	172.0	88	224.8	108
	300	318.5	106	275.0	91	313.5	90	279.6	93	242.7	81	251.7	86	287.5	103
river water	0	<LOD	-	<LOD	-	40.5	-	<LOD	-	<LOD	-	<LOD	-	68.0	
	100	104.8	105	91.3	91	146.8	106	85.6	86	89.8	90	104.8	105	137.0	103
	200	212.9	106	177.9	89	250.3	105	156.9	83	174.9	87	180.0	90	211.1	108
	300	316.5	106	276.3	92	331.3	97	281.2	93	246.3	82	261.8	87	273.3	103
tap water	0	<LOD	-	<LOD	-	74.4	-	<LOD	-	<LOD	-	<LOD	-	212.1	
	100	98.2	98	109.1	109	184.8	110	88.3	88	101.6	102	108.8	109	279.5	101
	200	210.3	105	178.0	90	281.6	104	159.5	80	175.3	88	170.2	86	333.1	91
	300	317.0	105	277.6	94	358.4	95	281.4	94	242.9	81	258.6	87	376.8	82

a Recovery (Rec %), limit of detection (LOD).

b Zn levels are 67, 133, and 200 μg L^–1^.

The accuracy experiments involved spiking real samples with a multielement standard solution containing 23 species (including alkali, alkaline earth, and other metals). Satisfactory recoveries were obtained for all target analytes across three spike levels (ranging from 81% to 109%), demonstrating that the presence of the other 16 ions and the sample matrix did not significantly interfere with the extraction process. These results indicate that the HDES-alginate system possesses sufficient selectivity for the target metals, effectively mitigating potential interference from common matrix components, such as chloride and sulfate, as well as from competing metal ions.

Additionally, the accuracy was corroborated by comparing the results to those obtained using an independent procedure (ICP-OES). Table 2 highlights the agreement between the two techniques for the quantifiable analytes (Cu and Zn), further validating the reliability of the proposed SPE procedure.

**Table 2:** Comparison of the Proposed Method Using FAAS with the Reference Method Using ICP-OES for Zn and Cu Determination in the Samples^a^

	proposed method (FAAS)				ICP-OES^b^	
**test sample**	Cu **(μg L** ^**–1**^ **)**	rec **(%)**	Zn **(μg L** ^**–1**^ **)**	rec **(%)**	Cu **(μg L** ^**–1**^ **)**	Zn **(μg L** ^**–1**^ **)**
lake water	38.3 ± 4.2	92	296.9 ± 11.7	92	41.5 ± 2.1	322.5 ± 21.4
rainwater	42.6 ± 2.1	108	80.9 ± 1.3	102	39.4 ± 3.6	79.7 ± 1.5
river water	40.5 ± 2.9	91	68.0 ± 2.1	109	44.3 ± 5.4	62.6 ± 1.5
tap water	74.4 ± 3.7	99	212.1 ± 19.8	93	75.2 ± 6.3	227.1 ± 31.5

a Recovery (REC).

b For Cd, Co, Fe, Mn, and Ni, < LOQ in analysis by ICP-OES.

The analytical performance parameters under the optimized conditions are summarized in Table 3. The method provided satisfactory enrichment factors for all analytes, effectively improving the sensitivity of FAAS determination. Precision was assessed via intraday and interday assays, yielding RSDs below 4.6% and 7.3%, respectively, which confirms the reproducibility of the extraction and determination steps.

**Table 3:** Analytical Parameters of the Proposed Procedure by Monitoring Each Element Individually through a Monoelemental Manner^a^

				RSD (%)	
analyte^b^	LOQ (μg L^–1^)	*R* ^2^	EF	intra-day	inter-day
Cd	1.7	0.9990	73	1.4	2.9
Co	9.0	0.9991	59	1.6	3.8
Cu	3.7	0.9982	67	1.0	3.8
Fe	7.3	0.9982	57	4.6	7.3
Mn	3.4	0.9998	59	0.9	3.3
Ni	12.6	0.9989	54	1.2	3.7
Zn	6.4	0.9992	69	1.4	3.2

a Limit of detection (LOD), limit of quantification (LOQ), enrichment factor (EF), and relative standard deviation (RSD).

b (Cd, Co, Fe, Mn, and Ni < LOQ in all samples).

Finally, Table 4 positions the developed method alongside other alginate-based extraction procedures, demonstrating its competitive performance in terms of the detection limits and enrichment factors.

**Table 4:** Comparison between Alginate-Based Extraction Methods for Elemental Determination in Water Samples by the Spectrometry Method^a^

sample	analyte	detection method	alginate system	extraction time (min)	linear range (μg L^–1^)	LOD (μg L^–1^)	EF	RSD (%)	reference
well and borehole water	Cd and Pb	ICP–MS	Ca-Alginate-P–ZrO_2_ CeO_2_ ZnO nanoparticles	n.d	0–1000	0.03–0.102 ng L^–1^	10	5.8–9.1	^20^
river and tap water	La, Ce, Pr, Nd, Sm, Eu, Gd, Tb, Dy, Ho, Er, Tm, Yb, and Lu	ICP–MS	Ca-alginate microparticles	30	n.d	0.01–0.03 ng kg^–1^	100	<4.0	^21^
groundwater	Ce, La, and Nd	ICP–OES	Ca alginate beads	120	1.0–10 mg L^–1^	n.d	20	n.d	^22^
tap water	Cu	FAAS	Ca-alginate microparticles	≈5	1.0–200 mg L^–1^	0.80	17	3.5	^23^
river water	Ni	FAAS	Ca-alginate-dimethylglyoxime microcapsules	n.d	50–1000	45.1	4	4.1	^24^
river water	Pb	FAAS	Ca-alginate beads	90	n.d	2.0	54	<5.0	^25^
river, lake, tap, and rainwater	Cd, Co, Cu, Fe, Mn, Ni, and Zn	FAAS	Ca-HDES-alginate beads	25	10–1000	0.5–3.8	73–54	<7.3	this work

a Inductively coupled plasma optical emission spectroscopy (ICP-OES), inductively coupled plasma mass spectrometry (ICP–MS), atomic absorption spectrometry (FAAS), hydrophobic deep eutectic solvent (HDES), limit of detection (LOD), limit of quantification (LOQ), enrichment factor (EF), relative standard deviation (RSD), and not informed (n.d.).

### 3.6 Applicability

Brazilian, American, and European regulations establish maximum permissible limits for various trace elements in drinking water, aiming to protect public health. In Brazil, these limits are defined by administrative rule GM/MS No. 888/2021; in the United States, the main reference is the Environmental Protection Agency (EPA) through the national primary drinking water regulations and secondary drinking water standards, and in the European Union, Directive (EU) 2020/2184 addresses the quality of water intended for human consumption. For Zn, Cd, Cu, Ni, Fe, and Mn, the regulatory limits vary, with Cd and Ni presenting the lowest limits due to their toxicity. Based on the LOD and LOQ achieved, the developed method demonstrates the capability to comply with the requirements of the regulations, enabling the determination of these analytes in water samples within their specified limits.^26–28^

## 4 Conclusions

The encapsulation of HDES in alginate capsules proved to be an innovative, sustainable, and highly effective strategy for the SPE of inorganic analytes. Beyond preserving the excellent extraction capability of HDES, the encapsulation approach successfully overcame major limitations typically associated with their direct application in liquid–liquid extraction coupled to spectrometric techniques, including spectral and nonspectral interferences, solvent accumulation, and nebulizer clogging. As a result, the proposed method significantly improved the precision, accuracy, and operational stability of FAAS measurements.

In addition to its analytical performance, the synthesis procedure based on the dripping method is straightforward, low-cost, and highly reproducible. Although performed at the laboratory scale using a peristaltic pump, the methodology presents strong potential for scale-up and adaptation to different analytical platforms and sample preparation workflows.

Overall, this work demonstrates that alginate-encapsulated HDESs represent a promising new generation of greener extraction materials, capable of combining high analytical performance with operational simplicity and environmental compatibility. The proposed strategy opens new perspectives for the development of sustainable sample-preparation methods for atomic spectrometry and other analytical applications.

## Supplementary Material



## Notes

The effect of the cross-linking ion on the extraction efficiency of the alginate-encapsulated HDES sorbent; the FTIR spectra of the sorbent; experimental matrix and multiple responses for the Box-Behnken design used to optimize the extraction step; the ANOVA for the quadratic models derived from both Box-Behnken and Doehlert experimental designs; experimental matrix and multiple responses for the Doehlert design applied to the desorption step optimization; and effect of sample volume on the recovery percentage of the analytes during the solid-phase extraction procedure (PDF)
