# Supplementary material for: Alginate-Encapsulated HDES as a Green Sorbent for Elemental Preconcentration Prior to Determination by FAAS
Source: ACS Omega. 2026 Jul 8;11(28):41860–9. doi: 10.1021/acsomega.6c01846 (PMC13393359; doi:10.1021/acsomega.6c01846)
Supplement: Supplementary file 1 [file ao6c01846_si_001.pdf]

Alginate-encapsulated HDES as a green sorbent for elemental preconcentration prior to determination by FAAS

Mateus Olivera Müller[1], Mariana E. M. Araújo[1], Adriano Lucas Paiva dos Santos[1], Floriatan Santos Costa[1], Marco Tadeu Grassi[1], Mario Henrique Gonzalez[2] and Clarice D. B. Amaral[1]*

1 Department of Chemistry, Federal University of Paraná, Curitiba, PR, 81531-980, Brazil

2 Department of Chemistry and Environmental Science, , São Paulo State University (UNESP), São José do Rio Preto, SP, 15054-000, Brazil

*E-mail: clariceamaral@ufpr.br

Supplementary information (SI)

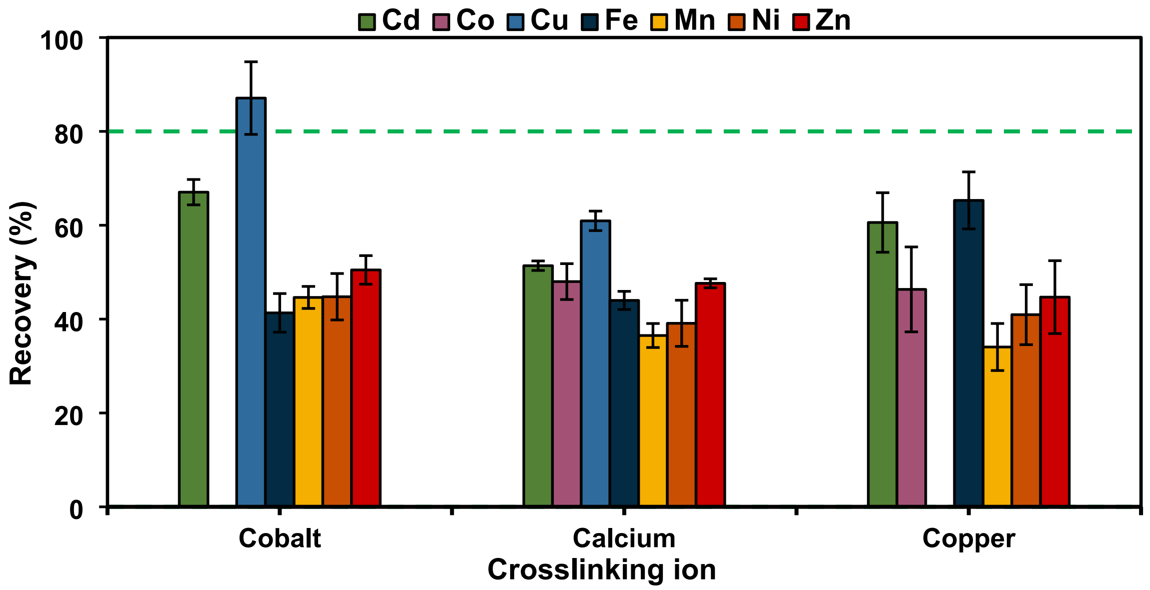

Figure S1 Effect of the crosslinking ion used in the encapsulation on the extraction efficiency of the method. All sorbents were made of Men:Cap with HDES/Alginate proportion of 25% v v^{-1}.

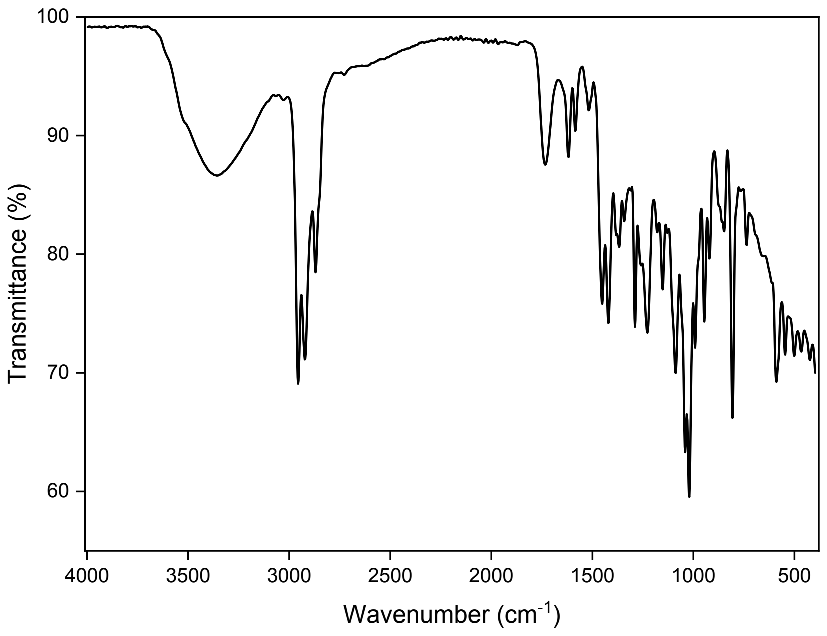

Figure S2 FTIR spectra of the alginate-HDES sorbent.

Table S1 Experimental matrix of the Box-Behnken design (real and coded levels) and multiple response used for the optimization of the extraction step of the proposed SPE method

Experiment	Sorbent mass – A (mg)	Extraction time – B (min)	Sample pH – C	Multiple response (MR)
1	200 (−1)	5 (−1)	4 (0)	1.64
2	1000 (1)	5 (−1)	4 (0)	5.28
3	200 (−1)	25 (1)	4 (0)	2.32
4	1000 (1)	25 (1)	4 (0)	6.52
5	200 (−1)	15 (0)	2 (−1)	1.24
6	1000 (1)	15 (0)	2 (−1)	4.82
7	200 (−1)	15 (0)	6 (1)	2.35
8	1000 (1)	15 (0)	6 (1)	6.74
9	600 (0)	5 (−1)	2 (−1)	2.70
10	600 (0)	25 (1)	2 (−1)	3.56
11	600 (0)	5 (−1)	6 (1)	3.70
12	600 (0)	25 (1)	6 (1)	5.92
13a	600 (0)	15 (0)	4 (0)	4.66
14a	600 (0)	15 (0)	4 (0)	4.33
15a	600 (0)	15 (0)	4 (0)	4.86

[a] Central point

Table S2 Analysis of variance for the quadratic model obtained using the Box-Behnken design (A) and Doehlert design (B)

**Design**	**Source of variation**	**SS**	**Df**	**MS**	**F-value**	***p*-value**
Box-Behnken design (A)	Regression	41.46	9	70.8	70.8	<0.0001*
(Quadratic model)[a]	Lack of Fit	0.18	3	0.06	0.9	0.5712**
	Pure Error	0.14	2	0.07		
	Total	41.78	14			
Doehlert design (B)	Regression	1.21	5	0.24	58.1	0.004*
(Quadratic model)[a]	Lack of Fit	0.01	1	0.01	14.0	0.065**
	Pure Error	0.00	2	0.00		
	Total	1.22	8			

Sum of squares (SS), degrees of freedom (Df), mean squares (MS)

[a] 95% confidence level

* Statistically significant ($p < 0.05$)

** Not Statistically significant ($p > 0.05$)

Table S3 Experimental matrix of the Doehlert design (real and coded levels) and multiple response used for the optimization of the desorption step of the proposed SPE method

Experiment	Desorption time – A (min)	HNO_3 – B (% v v^{-1})	Multiple response (MR)
1	9 (1)	3 (0)	6.07
2	7 (0.5)	5 (0.866)	6.50
3	1 (−1)	3 (0)	5.93
4	3 (−0.5)	1 (−0.866)	6.00
5	7 (0.5)	1 (−0.866)	6.45
6	3 (−0.5)	5 (0.866)	6.56
7a	5 (0)	3 (0)	6.88
8a	5 (0)	3 (0)	6.92
9a	5 (0)	3 (0)	6.87

[a] Central point

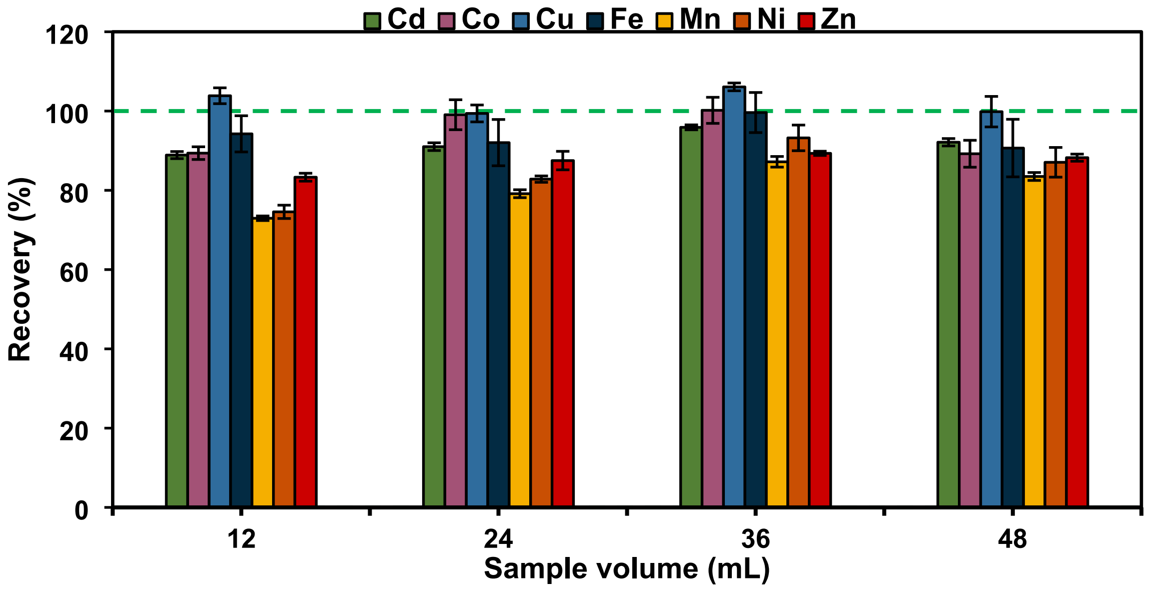

Figure S3 Effect of sample volume on the %recovery of analytes in the SPE procedure using the alginate-HDES sorbent.
